# The survival rate among endovascular and open surgical repair of abdominal aortic aneurysms

**DOI:** 10.1016/j.amsu.2021.102913

**Published:** 2021-10-05

**Authors:** Ahmed Abdel Rahim, Rashid Ibrahim, Lu Yao, Ahmed Khalf, Mohammed Ismail

**Affiliations:** a Plymouth University Hospital NHS Trust, United Kingdom; b Aswan University Hospitals, Egypt

**Keywords:** Infrarenal abdominal aortic aneurysm (AAA), Endovascular aneurysm repair (EVAR), Open surgical repair (OSR), Survival rates

## Abstract

A best evidence topic has been constructed using a described protocol. The three-part question addressed was: In patients with Infrarenal abdominal aortic aneurysm (AAA), Does endovascular abdominal aortic repair (EVAR), AS compared to open surgical repair (OSR), has higher Survival rates? The outcomes assessed were the overall survival rates in both techniques. The best evidence showed that there is no statistically significant difference between EVAR and OSR in survival rates.

## Highlights

• Endovascular abdominal aortic aneurysm repair is inferior to open surgical repair regarding the secondary rupture rates.
• Long term outcomes of the open surgical repair are better than the Endovascular repair.
• Secondary rupture is more common in endovascular repair than the open surgical repair of abdominal aortic aneurysm.

## 1 Introduction

This BET was designed using a framework outlined by the International Journal of Surgery [1]. This format was used because a preliminary literature search suggested that the available evidence is of insufficient quality to perform a meaningful meta-analysis. A BET provides evidence-based answers to common clinical questions, using a systematic approach of reviewing the literature.

## 2 Clinical scenario

While discussing the management options of 65 years old patient with Infrarenal abdominal aortic aneurysm (AAA) of 6.5 cm, one of the junior doctors asked which has better long term survival rate (>5 year); open surgical repair (OSR) or endovascular abdominal aortic repair (EVAR)?

Three Parts Question:

• [In patients with AAA,]
• [Which modality of treatment has higher long term overall survival rates];
• [EVAR or OSR]?

## 3 Search strategy

A Embase 1974 to June 2021 using the OVID interface:

[AAA OR Abdominal Aortic Aneurysm]AND [Open repair OR open surgical repair OR OSR] AND [EVAR OR Endovascular Repair] AND [Survival rate].

B. Medline using the PubMed interface:

[AAA OR Abdominal Aortic Aneurysm]AND [Open repair OR Open surgical repair OR OSR] AND [EVAR OR Endovascular Repair] AND [Survival rate].

The results were limited to English articles and human studies.

• Inclusion criteria: all original articles that review Survival rate among patients with AAA who underwent open surgical repair vs Endovascular Repair.
• Exclusion criteria: case reports, systematic reviews, letters to the editor, conference abstracts.

## 4 Search outcome

A total of 1741 papers were found using both search engines. We excluded 960 articles because they were irrelevant based on the titles and the abstracts. Seven hundred eighty-one full-text articles were screened and assessed for eligibility. From these, six papers were identified to provide the best evidence to answer the question. (see Table 1)

## 5 Result

**Table 1:** Summary of search results.

Author/date of publication/journal/country	Study type and level of evidence	Patient group	Outcomes follow up		Key results	Additional comments
Lederle F A et al., 2019, N Eng J Med, USA [2]. OVER	Randomized control trial- Level 1b	Total of 881 patients with AAA * Group 1 EVAR: 444 *Group 2 OSR: 437 *Follow-up, 14.2 years. *Median was 9.4 years	*End point is Overall survival rate. *Other outcomes: all cause or aneurysm related mortality, re-intervention rates and secondary ruptures		*<70 years old Group 1 EVAR: 45% Group 2 OSR: 41% *Hazard ratio for death, 0.81; 95% Confidence Interval (CI), 0.62–1.05. *P value = 0.10 **≥70 years old Group 1 EVAR: 19.5% Group 2 OSR: 21.7% *Hazard ratio for death, 1.20. *95% CI, 0.98–1.47 *P Value = 0.08 **P value for interaction = 0.02 *Statistically Insignificant	*Long term *Multi-Center *Specific skills and device training for the investigators.
Van Schaik et al., 2017, JVS, Netherland [3] Dream	Randomized controlled trial -level 1b	*Total of 351 patients with AAA *Group (1)OSR: 178 *Group (2) EVAR: 173 *Follow up was 12 years. * Median was 10.2 years.	*End point is Overall survival rate *Other outcomes: all-cause or aneurysm-related mortality and re-intervention rate.		Group 1 OSR: 42.2% Group 2 EVAR: 38.5% * (95% confidence interval, 6.7–14.1) *P-value = 0.48. *Statistically Insignificant	* Long term follow up *Multi-Center *Lack of Blinding. *Old devices used
Rajesh Patel et al., 2016, Lancet, UK [4]. EVAR-1	Randomized control trial- Level 1b	*Total of 1252 patients with AAA *Group 1 OSR: 626 *Group 2 EVAR: 626 * Follow-up:15.8 years. *Median was 12.4 years.	*End point is Overall survival rate. *Other outcomes: all-cause or aneurysm-related mortality and re-interventions.		*Group 1 OSR: 23.8%. *Group 2 EVAR: 14.8% *P value = 0.49 *Statistically Insignificant	* Long term *Multi Centre *Old devices used * Imaging was of low quality *Follow up changed from CT to Ultrasound .
Huang et al., 2015, JVS, USA [5].	Retrospective Cohort -Level 2a	*Total of 1116 patients with AAA *Group 1 OSR: 558 *Group 2 EVAR: 558 *Follow-up: 10 years; *Median was 7.6 years.	*End point is Overall survival rate *Other outcomes: all-cause or aneurysm-related mortality and re-interventions.		Group 1 OSR: 49% Group 2 EVAR: 33% * 95% confidence interval = 1.19–1.73; *P value < 0.001) *Statistically significant	*Large sample size *Retrospective *OSR group are less rigorously followed up.
Majd et al., 2017, Ann Vasc Surg, Germany [6].	Retrospective Cohort -Level 2a	*Total of 177 patients with AAA. *Group 1 EVAR: 131 (74%). *Group 2 OSR: 46 (26%). *Follow up: 7 years *Median was 5 years for the OSR group and 4.5 years for the EVAR group.	*End point is Overall survival rate. *Other outcomes: re-interventions, all-cause mortality	Five years Outcomes: *Group 1 EVAR: 38%. *Group 2 OSR: 50% *P-Value = 0.505 *Statistically insignificant		*Retrospective *Single-center *Small sample size * selection bias
Siracuse et al., 2016, Br J Surg, New England [7]	Retrospective Cohort-Level 2a	*Total of 1546 patients with AAA *Group 1 EVAR: 1070 Patients. *Group 2 OSR: 476 Patients *Follow up:11 years.	End point is Overall survival rate. *Other outcomes: re-interventions and all-cause mortality.	*Group 1 EVAR: 77.7% *Group 2 OSR: 72.8% *P value = 0.592. *Statistically insignificant		*Retrospective *Potentially biased data collection by institutions *Practice variations

## 6 Discussion

EVAR is no being considered as standard treatment for AAA because of the initial promising results [8].However, there are significant gaps in the evidence comparing the long-term results among patients of OSR and the EVAR [9].

In this article, we reviewed the best studies which compared the two modalities of AAA repair to evaluate which techniques have higher survival rates.

Only one study in our review showed a statistically significant difference between EVAR and OSR in survival rate in favor of the OSR group; this study was conducted by Huang et al. [5]. This study is retrospective with large sample size, but the OSR group is less rigorously followed up than the EVAR group. Also, most of the EVAR end grafts are no longer used now.

In contrast, there are another five trials, three of them were Randomized controlled trials which were conducted by Lederle FA et al. [2], Van Schaik et al. [3], and Rajesh Patel et al. [4], and another two retrospective cohort trials conducted by Majd et al. [6] and Siracuse et al. [7] show a statistically insignificant difference in survival rates among patients with OSR in comparison to EVAR. All of these studies included large sample size and long periods of follow-up.

## 7 Clinical bottom line

According to the above articles, the best evidence shows no statistically significant difference in long-term overall survival rate among patients with open surgical repair of abdominal aortic aneurysm compared to endovascular repair.

## Author contribution

Ahmed Abdel Rahim (AA): Conducted the literature search and wrote the paper.

Rashid Ibrahim (RI): Assisted in the literature search and Writing of paper.

Lu Yao(LY): Editing of writing.

Ahmed Khalf (AK): Assisted in writing of paper.

Mohammed Ismail (MI): Assisted in the literature search and writing of paper

## Ethical approval

Ethical approval was not required.

## Sources of funding

No source of funding.

## Registration of research studies

1. Name of the registry:

2. Unique Identifying number or registration ID:

3. Hyperlink to your specific registration (must be publicly accessible and will be checked):

## Guarantor

Ahmed Abdel Rahim.

## Consent

Ethics committee approval was not required as the study was review of previously done studies.

## Declaration of competing interest

No conflicts of interest.
